# *Sorting nexin 24* genetic variation associates with coronary artery aneurysm severity in Kawasaki disease patients

**DOI:** 10.1186/2045-3701-3-44

**Published:** 2013-11-22

**Authors:** Ying-Ju Lin, Jeng-Sheng Chang, Xiang Liu, Ting-Hsu Lin, Shao-Mei Huang, Chiu-Chu Liao, Cheng-Wen Lin, Wen-Kuei Chien, Jin-Hua Chen, Jer-Yuarn Wu, Chien-Hsiun Chen, Li-Ching Chang, Hsinyi Tsang, Kuan-Teh Jeang, Chia-Yen Chen, Fuu-Jen Tsai

**Affiliations:** 1 Department of Medical Research, China Medical University Hospital, Taichung, Taiwan; 2 School of Chinese Medicine, China Medical University, Taichung, Taiwan; 3 Department of Pediatrics, China Medical University Hospital, Taichung, Taiwan; 4 Molecular Virology Section, Laboratory of Molecular Microbiology, National Institute of Allergy and Infectious Diseases, National Institutes of Health, Bethesda, MD, USA; 5 Department of Medical Laboratory Science and Biotechnology, China Medical University, Taichung, Taiwan; 6 Biostatistics Center, China Medical University, Taichung, Taiwan; 7 Biostatistics Center, Taipei Medical University, Taipei, Taiwan; 8 Institute of Biomedical Sciences, Academia Sinica, Taipei, Taiwan; 9 The Laboratory of Molecular Immunogenetics, National Institute of Allergy and Infectious Diseases, National Institutes of Health, Bethesda, MD, USA; 10 Viral Biochemistry Section, Laboratory of Molecular Microbiology, National Institute of Allergy and Infectious Diseases, National Institutes of Health, Bethesda, MD, USA; 11 Asia University, Taichung, Taiwan

**Keywords:** Kawasaki disease, Coronary artery aneurysm, Sorting nexin 24, Polymorphism

## Abstract

**Background:**

The sorting nexin (SNX) family is involved in endocytosis and protein trafficking and plays multiple roles in various diseases. The role of SNX proteins in Kawasaki disease (KD) is not known. We attempted to test whether genetic *SNX* variation associates with the risk of coronary artery aneurysm (CAA) formation in KD.

**Methods and results:**

Chi-square tests were used to identify *SNX24* genetic variants associated with KD susceptibility and CAA formation in KD; models were adjusted for fever duration and time of first administration of intravenous immunoglobulin. We obtained clinical characteristics and genotypes from KD patients (76 with CAA and 186 without CAA) in a population-based retrospective KD cohort study (n = 262). Clinical and genetic factors were associated with CAA formation in KD. In addition, endothelial cell inflammation was evaluated. Significant correlation was observed between KD with CAA complications and the rs28891 single-nucleotide polymorphism in *SNX24*. Patients with CC + CT genotypes had lesser CAA complications. In lipopolysaccharide-treated human umbilical vein endothelial cells, siRNA knockdown of *SNX24* significantly decreased gene expression of the proinflammatory cytokines IL-1 beta, IL-6, and IL-8.

**Conclusions:**

Polymorphisms in *SNX24* may be used as genetic markers for the diagnosis and prognosis of CAA formation in KD.

## Background

Kawasaki disease (KD) is an acute and systemic vasculitis in children under 5 years old [1-5]. It is believed to be caused by infectious agents, host immune dysregulation, and genetic susceptibility. During the acute stage of KD, activation of vascular endothelial cells and increased serum levels of proinflammatory cytokines lead to inflammation and injury of blood vessels [6-8]. The vascular inflammation may induce the development of aneurysms and cardiac complications. Complications in coronary artery aneurysms (CAA) make KD one of the leading causes of acquired cardiovascular diseases in childhood. Until now, the pathological mechanism of CAA formation in KD has not been fully elucidated.

The sorting nexin (SNX) family of proteins consists of a diverse group of cytoplasmic or membrane-associated molecules that are characterized by the presence of a phospholipid-binding motif, the phox-homology (PX) domain, and are involved in endocytosis and protein trafficking [9,10]. The presence of a PX domain is the defining characteristic of this family and has been shown to bind various phosphatidylinositol phosphates (PtdInsPs) [11,12]. This domain is thought to lead these proteins to specialized membrane domains with specific phospholipids [13]. Sorting nexins have been associated with various diseases, including tumorigenesis, inflammation, pathogen infection, and Alzheimer disease [14-19]. The role of SNX proteins in endothelial cell injury and inflammation and their correlation with cardiovascular diseases such as KD remain to be elucidated.

To this end, we screened variants of 10 genes that encode SNX proteins associated with KD with CAA formation. The genes studied were *SNX17*, *SNX3*, *SNX10*, *SNX16*, *SNX22*, *SNX29*, *SNX20*, *SNX11*, *SNX21*, and *SNX24*. In this study, 262 KD patients (76 with CAA and 186 without CAA) were evaluated for clinical characteristics, the extent of aneurysm formation, and *SNX24* genotype. We observed that genetic variation in *SNX24* was associated with CAA formation in KD.

## Results

### Genotype frequencies of *SNX24* polymorphisms

A total of 262 KD patients and 1107 unrelated healthy control individuals were included in this study. The genotypes of the 9 genetic polymorphisms were identified by a custom-designed genotyping method [20]. The genotype frequencies of these polymorphisms are shown in Table 1. The KD patients did not differ from the control individuals with respect to the genotype frequencies of these polymorphisms (Table 1).

**Table 1:** **Genotype distributions of *SNX24 *** **gene SNPs in Taiwanese KD patients and controls**

**SNP**	**Chromosome**	**Cytoband**	**Physical position**	**Nearest genes**		**Controls**	**KD patients**		
						**No. (%)**	**No. (%)**	***p *** **value**	**Odds ratio (95% CI)**
rs154507	5	q23.2	122228806	*SNX24*	GG	146 (13.2)	38 (14.5)	0.755	1.17 (0.76–1.78)
					GC	553 (50.0)	133 (50.8)		1.08 (0.80–1.45)
					CC	408 (36.8)	91 (34.7)		1
rs27740	5	q23.2	122232671	*SNX24*	TT	156 (14.1)	38 (14.5)	0.867	1.08 (0.71–1.65)
					TA	547 (49.4)	133 (50.8)		1.08 (0.80–1.45)
					AA	404 (36.5)	91 (34.7)		1
rs26371	5	q23.2	122234424	*SNX24*	TT	217 (19.6)	56 (21.4)	0.690	1.19 (0.80–1.76)
					TC	573 (51.8)	137 (52.3)		1.10 (0.80–1.51)
					CC	317 (28.7)	69 (26.3)		1
rs6595415	5	q23.2	122234657	*SNX24*	TT	151 (13.6)	38 (14.5)	0.842	1.12 (0.73–1.70)
					TC	530 (47.9)	128 (48.9)		1.07 (0.80–1.44)
					CC	426 (38.5)	96 (36.6)		1
rs17149732	5	q23.2	122249811	*SNX24*	TT	146 (13.2)	37 (14.1)	0.784	1.14 (0.74–1.73)
					TG	530 (47.9)	129 (49.2)		1.09 (0.81–1.46)
					GG	430 (38.9)	96 (36.6)		1
rs17149748	5	q23.2	122295266	*SNX24*	CC	150 (13.6)	37 (14.1)	0.710	1.13 (0.74–1.72)
					CT	532 (48.1)	131 (50.0)		1.13 (0.84–1.51)
					TT	425 (38.4)	93 (35.5)		1
rs1038078	5	q23.2	122309550	*SNX24*	AA	147 (13.3)	37 (14.1)	0.676	1.15 (0.75–1.76)
					AG	526 (47.5)	130 (49.6)		1.13 (0.84–1.51)
					GG	434 (39.2)	95 (36.3)		1
rs28891	5	q23.2	122311523	*SNX24*	CC	187 (16.9)	47 (17.9)	0.836	1.12 (0.75–1.67)
					CT	554 (50.1)	133 (50.8)		1.07 (0.79–1.45)
					TT	366 (33.1)	82 (31.3)		1
rs6595423	5	q23.2	122341433	*SNX24*	CC	144 (13.0)	36 (13.7)	0.667	1.14 (0.75–1.75)
					CT	524 (47.3)	130 (49.6)		1.13 (0.85–1.52)
					TT	439 (39.7)	96 (36.6)		1

*SNX24*, sorting nexin 24; SNP, single nucleotide polymorphism; CI, confidence interval.

*p*-values were obtained by chi-square test.

Bold, emphasizing statistical significance was considered as *p* value <0.006 (0.05/9).

### *SNX24* single-nucleotide polymorphism rs28891 is associated with CAA formation in KD

**Figure 1:**
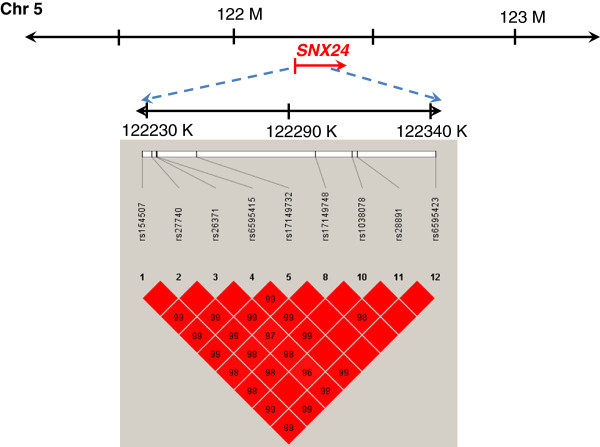
**Analysis of the single-nucleotide polymorphisms (SNPs) used in this study, and the linkage disequilibrium (LD) pattern of the *SNX24 *gene.** The genomic location of SNPs present on chromosome 5q23.2. LD blocks in the *SNX24* gene were estimated by using HAPLOVIEW. Pairwise D’ values (%, n = 27) are indicated in squares; red indicates linkage disequilibrium (D’ = 1, logarithm of odds (LOD) ≥ 2).

Subsequently, we genotyped the 262 KD patients for *SNX24* single-nucleotide polymorphisms (SNPs) (Table 2). The genetic location of *SNX24* is shown in Figure 1; the frequency of genotyping success was >99%. The linkage disequilibrium (LD) structure of this region was also established and was found to consist of a single haplotype block.

**Table 2:** Summary of the SNPs associated with the CAA formation in Taiwanese Kawasaki disease

**SNP**	**Chromosome**	**Cytoband**	**Physical position**	**Nearest genes**		**CAA-**	**CAA+**		
						**No. (%)**	**No. (%)**	***p *** **value**	**Odds ratio (95% CI)**
rs154507	5	q23.2	122228806	*SNX24*	GG	24 (12.9)	14 (18.4)	0.234	1.94 (0.86–4.41)
					GC	92 (49.5)	41 (54.0)		1.49 (0.81–2.74)
					CC	70 (37.6)	21 (27.6)		1
rs27740	5	q23.2	122232671	*SNX24*	TT	24 (12.9)	14 (18.4)	0.234	1.94 (0.86–4.41)
					TA	92 (49.5)	41 (54.0)		1.49 (0.81–2.74)
					AA	70 (37.6)	21 (27.6)		1
rs26371	5	q23.2	122234424	*SNX24*	TT	44 (23.7)	12 (15.8)	0.123	0.45 (0.20–1.01)
					TC	99 (53.2)	38 (50.0)		0.63 (0.34–1.17)
					CC	43 (23.1)	26 (34.2)		1
rs6595415	5	q23.2	122234657	*SNX24*	TT	23 (12.4)	15 (19.7)	0.198	2.07 (0.93–4.61)
					TC	90 (48.4)	38 (50.0)		1.34 (0.73–2.45)
					CC	73 (39.2)	23 (30.3)		1
rs17149732	5	q23.2	122249811	*SNX24*	TT	23 (12.4)	14 (18.4)	0.261	1.93 (0.86–4.36)
					TG	90 (48.4)	39 (51.3)		1.38 (0.75–2.51)
					GG	73 (39.2)	23 (30.3)		1
rs17149748	5	q23.2	122295266	*SNX24*	CC	23 (12.4)	14 (18.4)	0.242	1.96 (0.87–4.45)
					CT	91 (49.2)	40 (52.6)		1.42 (0.77–2.60)
					TT	71 (38.4)	22 (29.0)		1
rs1038078	5	q23.2	122309550	*SNX24*	AA	23 (12.4)	14 (18.4)	0.283	1.91 (0.84–4.30)
					AG	91 (48.9)	39 (51.3)		1.34 (0.74–2.45)
					GG	72 (38.7)	23 (30.3)		1
rs28891	5	q23.2	122311523	*SNX24*	CC	39 (21.0)	8 (10.6)	***0.006***	0.29 (0.12–0.70)
					CT	99 (53.2)	34 (44.7)		0.48 (0.27–0.87)
					TT	48 (25.8)	34 (44.7)		1
rs6595423	5	q23.2	122341433	*SNX24*	CC	23 (12.4)	13 (17.1)	0.322	1.79 (0.79–4.10)
					CT	90 (48.4)	40 (52.6)		1.41 (0.78–2.57)
					TT	73 (39.2)	23 (30.3)		1

SNX24, sorting nexin 24; SNP, single nucleotide polymorphism; CAA, Coronary artery aneurysm; CI, confidence interval.

p-values were obtained by chi-square test.

Bold, emphasizing statistical significance was considered as *p* value <0.05.

Genotype and genotype frequency data for all 9 SNPs are shown in Table 2. A statistically significant difference was observed for the *SNX24* (rs28891) genetic variant (*p* = 0.006). The frequencies of individuals carrying the CC and CT genotypes of *SNX24* (rs28891) were 10.6% and 44.7%, respectively, for CAA-positive individuals. The frequencies of the CC and CT genotypes were significantly lower in KD individuals with CAA formation (CC genotype: odds ratio (OR) = 0.29, 95% confidence interval (CI) = 0.12–0.70; CT genotype: odds ratio = 0.48, 95% CI = 0.27–0.87) compared to KD individuals without CAA formation.

### Associating variation in the *SNX24* SNP rs28891 to CAA formation in KD by using logistic regression analysis

To determine the genetic role of *SNX24*, we used logistic regression analysis to rule out the clinical potential factor effects in CAA formation in KD. As shown in Table 3, after adjusting for these factors [21-25], there was a significant association between the *SNX24* SNP rs28891 and the incidence of KD with CAA. Specifically, significant correlations were found between KD with CAA formation and the CC + CT genotypes of the *SNX24* SNP rs28891 (model adjusted by fever duration: odds ratio = 0.41, 95% CI = 0.25–0.75, *p* = 0.003; model adjusted by time of first administration of intravenous immunoglobulin (IVIG): odds ratio = 0.42, 95% CI = 0.25–0.75, *p* = 0.002).

**Table 3:** **Association of *SNX24 *** **genetic variants with CAA formation risk in Taiwanese Kawasaki disease by logistic regression analysis**

***SNX24 *** **genetic variants**	**Odds ratio**	**95% CI**	***p *** **value**
**Adjusted by fever duration (days)**			
rs154507	1.90	0.88-2.83	0.046
rs27740	1.90	0.88-2.83	0.046
rs26371	0.60	0.32-1.04	0.105
rs6595415	1.74	0.84-2.64	0.077
rs17149732	1.74	0.84-2.64	0.077
rs17149748	1.83	0.86-2.72	0.057
rs1038078	1.71	0.82-2.58	0.087
rs28891	0.41	0.25-0.75	***0.003***
rs6595423	1.74	0.84-2.64	0.077
**Adjusted by 1st IVIG used (days after the first date with fever)**			
rs154507	1.61	0.88-2.83	0.114
rs27740	1.61	0.88-2.83	0.114
rs26371	0.57	0.32-1.04	0.059
rs6595415	1.52	0.84-2.64	0.152
rs17149732	1.52	0.84-2.64	0.152
rs17149748	1.57	0.86-2.72	0.127
rs1038078	1.50	0.82-2.58	0.171
rs28891	0.42	0.25-0.75	***0.002***
rs6595423	1.52	0.84-2.64	0.152

*SNX24*, sorting nexin 24; IVIG, Intravenous immunoglobulin; CAA, Coronary artery aneurysm; CI, confidence interval.

A logistic regression model was performed by using the indicated predictors including fever duration (days) or 1st IVIG used time (days after the first date with fever).

Bold, emphasizing statistical significance was considered as p value <0.006 (0.05/9).

**Figure 2:**
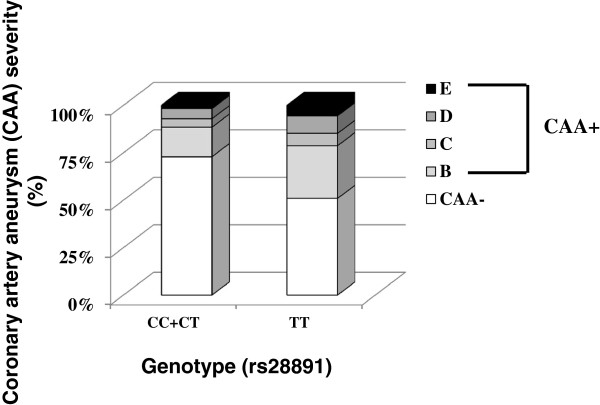
**Genotype distribution (*SNX24 *rs28891) in relation to coronary artery aneurysm (CAA) severity in the study population.** CAA severity grade: CAA- indicates patients with no complication; CAA + B grade indicates patients with CAAs at the beginning but that are in remission within 2 months; CAA + C grade indicates patients with CAAs that persist for 2 months but that are in remission within 6 months; CAA + D grade indicates patients with CAAs that persist for 6 months; CAA + E grade indicates patients with giant CAAs (≥8 mm) or severe stenosis or occlusion.

The genotype distributions of rs28891 in relation to CAA severity in the study population are also shown in Figure 2. As shown in Figure 2, there were lower percentages of the *SNX24* SNP genotypes CC + CT in patients with CAA formation compared to patients with the TT genotype; this suggests that patients with the CC + CT genotypes were correlated with less severe CAA complications.

### Inhibition of *IL-1 beta*, *IL-6*, and *IL-8* mRNA expression by downregulation of *SNX24* in lipopolysaccharide-treated human umbilical vein endothelial cells

**Figure 3:**
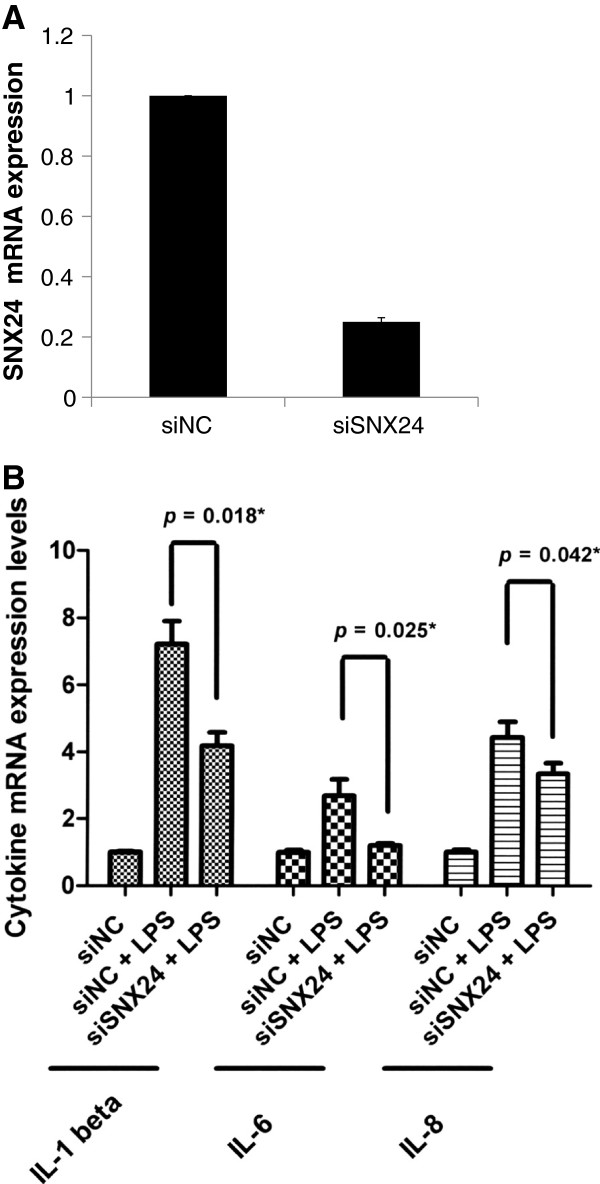
**Effect of *SNX24 *knockdown on proinflammatory cytokines.** HUVECs were transfected with siSNX24 or siNC for 24 h at 37°C followed by 100 μg/mL LPS for another 24 h. **A.** siSNX24 knockdown efficacy in HUVECs by using RT-qPCR assay. **B.***IL-1 beta*, *IL-6*, and *IL-8* mRNA expression was quantified by RT-qPCR. * *p* < 0.05 versus siNC + LPS. Data represent mean ± SD for 3 independent experiments.

To evaluate the effect of *SNX24* on endothelial cell inflammation, we used lipopolysaccharide (LPS) (an integral part of the outer membrane of gram-negative bacteria) as a pathogenic stimulus. Human umbilical vein endothelial cells (HUVECs) were transfected with short interfering RNAs (siRNAs) and then were treated with LPS. The proinflammatory cytokine expression in the endothelial cells was then analyzed by using real-time qPCR assays (Figure 3). HUVECs were transiently transfected with siRNAs targeting SNX24 (siSNX24), and the effect was assessed (Figure 3A). Compared to siNC-transfected HUVECs, cells transfected with siSNX24 resulted in a significant decrease in *SNX24* mRNA expression (Figure 3A). In addition, siRNA-transfected cells were then exposed to LPS. As shown in Figure 3B, treatment of HUVECs with LPS significantly increased the expression of *IL-1 beta*, *IL-6*, and *IL-8*. siRNA knockdown of *SNX24* significantly decreased gene expression of the proinflammatory cytokines IL-1 beta, IL-6, and IL-8, suggesting that *SNX24* may regulate endothelial cell inflammation.

## Discussion

To the best of our knowledge, no study to date has investigated the possible association of *SNX* family genes and the CAA formation in Kawasaki disease. In this study, we screened genetic variants of 9 SNX genes in relation to KD with CAA formation and identified a SNP in *SNX24* that correlates with the development of CAA formation in Taiwanese children of Han Chinese ethnic background with KD. We observed a significant association between the *SNX24* SNP and the occurrence of CAA in KD patients by using a logistic regression analysis. The frequencies of the CC and CT genotypes of *SNX24* SNP rs28891 were lower in the group with KD and CAA than in the group with KD but without CAA. Cell inflammatory activity was inhibited in siSNX24-treated cells. Our results suggest that polymorphisms in *SNX24* may be used as genetic markers for the diagnosis and prognosis of the CAA formation in KD.

Our results showed that the increased CAA formation in KD was associated with clinical risk factors, including fever duration and the time of administering the first IVIG treatment (days after the first incidence of fever). These findings also correspond with those reported previously in patients with KD [21-25]. Longer fever duration and the delay of IVIG treatment seem to be risk factors for the development of CAA in patients with KD. Prolonged fever duration suggests a state of longer and more severe inflammation. Endothelial cell injury and inflammations are known to be the main mechanisms in the development of KD [4]. When endothelial cells are stimulated with pathogenic mediator LPS, inflammatory signals are triggered, which increase permeability and leukocyte recruitment [26]. KD is a multi-systemic disorder with immune-mediated vasculitis and is very likely to influence CAA complications.

This genetic association study showed that significant associations between KD with CAA formation and the *SNX24* SNP rs28891 were still observed by using logistic regression analysis. The frequency of genotypes with 1 or 2 copies of the C allele were lower in KD patients with CAA formation. This SNP is located in the intron region of *SNX24*. Intronic SNPs may have an impact on splicing efficiency, the stability of the transcribed mRNA, and enhancer activity [27]. Change in the secondary structure of the pre-mRNA by a nucleotide substitution may influence splicing and mRNA formation [28,29]. In addition, the SNP we identified (rs28891) exhibited LD with the SNP (rs1038078) (Additional file 1: Table S1; D’ = 1). *SNX24* expression has been shown to be significantly associated with the SNP (rs1038078) (*p* = 0.04329) in the Han Chinese population (http://app3.titan.uio.no/biotools/tool.php?app=snpexp). Therefore, it is likely that the CC and CT genotypes of the *SNX24* gene polymorphism may be associated with transcript abundance. To further investigate the functional consequences of *SNX24* polymorphism, we first investigated the correlation between the SNP rs28891 genotypes and SNX24 expression (Additional file 2: Figure S1). We measured *SNX24* mRNA levels by real-time quantitative PCR in peripheral blood mononuclear cells. As shown, the major allele homozygotes (TT genotype) tended to express higher levels of *SNX24* than did the other individuals with CC + CT genotype (*p* = 0.035; Additional file 2: Figure S1). Our results also suggest that the frequencies of CC and CT genotypes were significantly lower in KD with CAA formation compared to those in KD without CAA formation. A possible explanation is that the SNX24 protein expression levels are involved in inflammations of vascular endothelial cells and the consequences of CAA complications in KD patients.

Endothelial cell injury and inflammations are known to be the main mechanisms in the development of KD [4]. When endothelial cells are stimulated with pathogenic mediator LPS, the stimulated cells trigger inflammatory signals to increase permeability and leukocyte recruitment [26]. In this study, we used this LPS-induced endothelial cell inflammation model and showed, for the first time, that *SNX24* may regulate endothelial cell inflammation by interfering with IL-1 beta, IL-6, and IL-8 expressions. *SNX24* is located on chromosome 5q23.2. It is preferentially expressed in platelets and blood plasma (http://www.genecards.org/cgi-bin/carddisp.pl?gene=SNX24&search=SNX24#diseases). Estrogen has been found to significantly upregulate the expression of SNX24 in certain breast cancer cell lines, suggesting that it has a role in tumorigenesis [30]. Conversely, SNX family proteins have been linked to trafficking and cell surface presentation of adhesion molecules on leucocytes, platelets, and endothelial cells. SNX family proteins might be involved in vasculitis and endothelial cell pathogenesis. Another member in the SNX family, SNX17, was identified as a P-selectin-interacting protein. It can decrease the degradation of P-selectin in lysosomes by restricting its transport into late endosomes, thereby affecting leucocyte recruitment [15,31]. The release of chemokines and cytokines by macrophages within injured tissue will initiate a series of events whereby leucocytes are attached to the endothelium [32,33].

We screened genetic variants of 9 genes that encode *SNX* genes linked to KD with CAA formation (Additional file 1: Table S1). These genes include *SNX17*, *SNX3*, *SNX10*, *SNX16*, *SNX22*, *SNX29*, *SNX20*, *SNX11*, and *SNX21*. This gene family encodes proteins that belong to a diverse group of cytoplasmic and membrane-associated proteins and are involved in various aspects of endocytosis and protein trafficking through membranous cellular compartments [9]. No significant statistical difference was observed for these SNX genes with respect to KD with CAA formation. We only observed a significant association between KD with CAA formation and the *SNX24* (rs28891). Therefore, we suggest that this *SNX24* genetic variant has a role in CAA formation in KD patients. In addition, in our study of the functional significance of *SNX24* in endothelial cell inflammation, we used siRNA knockdown of *SNX24* to significantly decrease gene expression of the proinflammatory cytokines of *IL-1 beta*, *IL-6*, and *IL-8*. This is the first study to report that *SNX24* is a regulator of vascular inflammation and may be beneficial for many inflammatory diseases associated with endothelial dysfunction.

## Methods

### Study subjects

Unrelated individuals fulfilling the diagnostic criteria of KD (n = 262) were identified and enrolled in the study from the Department of Pediatrics at China Medical University Hospital in Taichung, Taiwan [21,34-37]. All patients were diagnosed according to KD criteria [21,35], including fever lasting 5 days or more and at least 4 of the following symptoms: (1) changes in extremities (e.g., erythema, edema, or desquamation), (2) bilateral conjunctivitis, (3) polymorphous rash, (4) cervical lymphadenopathy, and (5) changes in the lips or oral cavity (e.g. pharyngeal erythema, dry/fissured or swollen lips, or “strawberry tongue”). All patients received IVIG treatment and had regular echocardiographic examinations during the 1-year follow-up. The echocardiographic examinations were made during the acute stage, 2 months after onset, 6 months after onset, and once per year thereafter. CAA was identified when either the right or the left coronary artery showed a dilated diameter ≥3 mm in children younger than 5 years of age, or ≥4 mm in older children [38]. Only Han Chinese individuals, who account for 98% of Taiwanese residents, were recruited. The ethnic background was assigned based on the results of self-reported questionnaires. This study was approved by the Human Studies Committee of China Medical University Hospital. Written informed consent was obtained from either the parents or the participants.

### SNP genotyping

Nine SNPs located in *SNX24* were randomly selected from the identified candidate SNPs that conformed to a set of criteria, by using the HAPLOVIEW software (Figure 1 and Table 2) [39-41]. The selection criteria that were used included a minimum allele frequency of *p* > 0.05 in the Han Chinese population, and no deviation from HWE (*p* > 0.05). A summary of information regarding the *SNX24* SNPs (location, position, rs number, and genotype) is listed in Table 2. Briefly, genomic DNA was extracted from peripheral blood leukocytes according to standard protocols (Genomic DNA kit; Qiagen, Hilden, Germany). SNPs were genotyped using a custom-designed VeraCode GoldenGate Genotyping Assay System (Illumina, San Diego, CA, USA) [42]; genotyping was performed as described at http://www.illumina.com/.

Primers and probes were designed using the Custom VeraCode GoldenGate Genotyping Assay System software. Multiplex PCRs were performed with 144-plex VeraCode SNP arrays (Illumina) using these samples, and genotype analyses were performed using custom 96-plex SAM arrays for 96 samples. Genotype calls were automatically generated using GenCall version 3.1.3. We assessed 8 VeraCode runs individually for intra-plate inconsistencies, such as variation in the intensity of the fluorescence. Genotype cluster plots generated from individual VeraCode and SAM assays were visually inspected for call quality. Plots that appeared to be “unusually” clustered (i.e., those that did not match the predicted spread in terms of software-generated HWE or distance between clusters [θ]) were investigated further by confirming the genotype of the selected samples via direct Sanger sequencing. Samples were sequenced using Big Dye Terminator v3.1 (AB, Foster City, CA, USA) according to the manufacturer’s guidelines and sequenced using a 3730 genetic analyzer (Life Technologies, Carlsbad, CA, USA).

### Analysis of haplotype blocks

Based on HAPLOVIEW, we used Lewontin’s D’ to estimate the inter-marker coefficient of LD of patients [41]. The confidence interval of LD was estimated using a resampling procedure and was used to construct haplotype blocks [43,44].

### Statistical analysis

Unless otherwise indicated, data are expressed as the mean ± SD for continuous variables. The unpaired Student’s *t* test was used to compare groups. Genotypes were obtained by direct counting followed by allele frequency calculations (Table 2). Chi-squared tests were used to identify differences in categorical variables, and OR and 95% CI were calculated for the factors under consideration. Forward stepwise multivariate regression analyses were also performed to identify factors contributing independently to CAA formation in KD. All statistical analyses were performed using SPSS v12.0 for Windows (IBM, Armonk, NY, USA).

### Cells

HUVECs (BCRC Number: H-UV001) were grown in 90% GIBCO medium 199 (Life Technologies) with 25 U/mL heparin (Sigma), 30 μg/mL endothelial cell growth supplement (Millipore) adjusted to contain 1.5 g/L sodium bicarbonate + 10% fetal bovine serum and 100 U/mL penicillin/streptomycin.

### Short interfering RNA

siRNAs targeting transcripts for *SNX24* (siSNX24: CAGAAAUCCCUUCUAAACAUGUUAG) were purchased from Invitrogen, as was the non-targeting siRNA control (scrambled (siNC: duplex 1, AUGAACGUGAAUUGCUCAA; duplex 2, UAAGGCUAUGAAGAGAUAC; duplex 3, AUGUAUUGGCCUGUAUUAG; duplex 4, UAGCGACUAAACACAUCAA)).

### Endothelial cell inflammation assay

For endothelial cell inflammation assay, HUVECs were aliquoted in 6-well plates. Cells were transfected by either siNC or siSNX24 using Lipofectamine 2000 (Invitrogen). The transfected cells were then treated with 100 μg/mL LPS for another 24 h. Cellular RNA extraction and real-time RT-PCR analyses were performed.

### Real-time RT-PCR analyses

HUVECs transfected by either siNC or siSNX24 were incubated for 36 h at 37°C. Cellular RNA isolation was performed using a QIAamp® RNA Mini Kit according to the manufacturer’s instructions (Qiagen, Valencia, CA, USA). RNA was eluted in 60 μL of buffer, and real-time TaqMan RT-PCR assays were used to determine the effects of siSNX24 knock down. The primers used to amplify *SNX24* by qPCR were 5′ forward primer: 5′-CGTCCTTTCGCTATGAAGAGA-3′ and 3′ reverse primer: 5′-TTCTTCCATTCATTAGCACTTCTATC-3′. The primers for *IL-1 beta* qPCR were 5′ forward primer: 5′-tacctgtcctgcgtgttgaa-3′ and 3′ reverse primer: 5′-tctttgggtaatttttgggatct-3′. The primers for *IL-6* qPCR were 5′ forward primer: 5′-caggagcccagctatgaact-3′ and 3′ reverse primer: 5′-gaaggcagcaggcaacac-3′. The primers for *IL-8* qPCR were 5′ forward primer: 5′-gagcactccataaggcacaaA-3′ and 3′ reverse primer: 5′-atggttccttccggtggt-3′. Reverse transcription was performed in a 10-μL reaction mixture consisting of 2 μL RNA template, 1 μL RT primer mix, 1 μL dNTP mix (10 mM of each), and 6 μL of RNA/DNAse-free water, at 65°C for 5 min. Next, a reaction mixture of 4 μL 5× MMLV buffer, 0.8 μL MMLV enzyme, and 5.2 μL RNA/DNAse-free water was added to each RNA sample. Reverse transcription reactions were performed at 42°C for 60 min. cDNA was amplified by PCR in a 20-μL reaction mixture containing 5 μL cDNA, 10 μL, 2× Mastermix, 1 μL primer/probe mix, and 4 μL RNA/DNAse-free water. Real-time TaqMan RT-PCR conditions were 95°C for 10 min, 50 cycles of 95°C for 10 s, and 60°C for 60 s. *SNX24* RNA levels were detected using a 7900HT Fast Real-Time PCR System (Life Technologies).

## Competing interest

The authors declare that they have no competing interest.

## Authors’ contributions

YJL, JSC, XL and FJT conceived and designed the experiments. THL, SMH, CCL, CWL and HT performed the experiments. WKC and JHC analyzed the data. JSC, JYW, CHC, LCC, KTJ and CYC contributed reagents/materials/analysis tools. YJL and XL wrote the manuscript. All authors read and approved the final manuscript.

## Supplementary Material

Additional file 1: Table S1 Summary of the SNPs from *SNX* gene family associated with the CAA formation in Taiwanese Kawasaki disease. **Table S2.** Haplotype distributions of *SNX24* gene SNPs associated with the CAA formation in Taiwanese KD patients. **Table S3.** Genotype distributions of *SNX24* gene SNPs in Taiwanese male and female KD patients. **Table S4.** Genotype distributions of *SNX24* gene SNPs in Taiwanese male KD patients. **Table S5.** Genotype distributions of *SNX24* gene SNPs in Taiwanese female KD patients. Click here for file

Additional file 2: Figure S1 *SNX24* mRNA expression levels in peripheral blood mononuclear cells between the *SNX24* SNP (rs28891) genotypes. The relative *SNX24* expression was detected by real-time RT-PCR, and expression from individuals with CC + CT genotypes was compared to that from individuals with TT genotypes. The relative expression levels were expressed as *SNX24* mRNA/ *HPRT* mRNA ratio. **Figure S2.** Single nucleotide polymorphisms (SNPs) of the *SNX24* gene used in this study. Above and middle: Genomic location of SNPs present on chromosome 5. Down: Non-coding RNAs mapped to the intron 3 of *SNX24* gene. Click here for file
