# Supplementary material for: Sorting nexin 24 genetic variation associates with coronary artery aneurysm severity in Kawasaki disease patients
Source: Cell Biosci. 2013 Nov 22;3:44. doi: 10.1186/2045-3701-3-44 (PMC4176999; doi:10.1186/2045-3701-3-44)
Supplement: Additional file 1: Table S1 — Summary of the SNPs from SNX gene family associated with the CAA formation in Taiwanese Kawasaki disease. Table S2. Haplotype distributions of SNX24 gene SNPs associated with the CAA formation in Taiwanese KD patients. Table S3. Genotype distributions of SNX24 gene SNPs in Taiwanese male and female KD patients. Table S4. Genotype distributions of SNX24 gene SNPs in Taiwanese male KD patients. Table S5. Genotype distributions of SNX24 gene SNPs in Taiwanese female KD patients. [file 2045-3701-3-44-S1.pdf]

Table S1 Summary of the SNPs from *SNX* gene family associated with the CAA formation in Taiwanese Kawasaki disease

SNP	Chromosome	Cytoband	Physical position	Nearest genes		CAA-	CAA+		
						No. (%)	No. (%)	*p* value	Odds ratio (95% CI)
rs2280737	2	p23.3	27443314	*SNX17*	GG	4 (2.2)	3 (4.0)	0.665	1.90 (0.41-8.76)
					GA	44 (24.2)	20 (26.3)		1.15 (0.62-2.13)
					AA	134 (73.6)	53 (69.7)		1
rs17006049	2	p23.3	27465746	*SNX17*	AA	0 (0.0)	0 (0.0)	0.861	ND
					AG	26 (14.0)	10 (13.2)		0.93 (0.43-2.04)
					GG	160 (86.0)	66 (86.8)		1
rs217132	6	q21	108662892	*SNX3*	AA	4 (2.1)	1 (1.3)	0.878	0.62 (0.07-5.63)
					AG	34 (18.3)	15 (19.7)		1.09 (0.55-2.14)
					GG	148 (79.6)	60 (79.0)		1
rs3800223	6	q21	108679188	*SNX3*	AA	27 (14.5)	17 (22.4)	0.300	1.74 (0.82-3.71)
					AT	87 (46.8)	33 (43.4)		1.05 (0.58-1.92)
					TT	72 (38.7)	26 (34.2)		1
rs9398166	6	q21	108686901	*SNX3*	GG	6 (3.3)	2 (2.6)	0.519	0.89 (0.17-4.57)
					GT	46 (24.7)	24 (31.6)		1.40 (0.77-2.52)
					TT	134 (72.0)	50 (65.8)		1
rs972158	7	p15.2	26301532	*SNX10*	AA	19 (10.2)	7 (9.2)	0.902	0.85 (0.33-2.19)
					AC	82 (44.1)	32 (42.1)		0.90 (0.51-1.57)
					CC	85 (45.7)	37 (48.7)		1
rs12671122	7	p15.2	26335810	*SNX10*	AA	5 (2.7)	5 (6.7)	0.136	2.98 (0.82-10.8)
					AC	59 (31.7)	29 (38.7)		1.46 (0.83-2.58)
					CC	122 (65.6)	41 (54.6)		1
rs10252532	7	p15.2	26346177	*SNX10*	CC	32 (17.2)	24 (31.6)	0.036	2.21 (1.03-4.73)
					CT	104 (55.9)	35 (46.1)		0.99 (0.51-1.94)
					TT	50 (26.9)	17 (22.4)		1
rs3801890	7	p15.2	26352679	*SNX10*	GG	34 (18.4)	24 (31.6)	0.066	1.99 (0.93-4.27)
					GA	103 (55.7)	35 (46.0)		0.96 (0.49-1.88)
					AA	48 (25.9)	17 (22.4)		1
rs1534696	7	p15.2	26363764	*SNX10*	CC	6 (3.2)	1 (1.3)	0.684	0.40 (0.05-3.40)
					CA	58 (31.2)	24 (31.6)		0.99 (0.56-1.76)
					AA	122 (65.6)	51 (67.1)		1
rs1463259	8	q21.13	82900300	*SNX16*	GG	19 (10.2)	10 (13.1)	0.208	1.78 (0.73-4.37)
					GA	89 (47.9)	43 (56.6)		1.64 (0.91-2.96)
					AA	78 (41.9)	23 (30.3)		1
rs12677458	8	q21.13	82900341	*SNX16*	TT	3 (1.6)	2 (2.7)	0.724	1.76 (0.29-10.81)
					TC	44 (23.9)	20 (27.0)		1.20 (0.65-2.22)
					CC	137 (74.5)	52 (70.3)		1
rs7164156	15	q22.31	62226620	*SNX22*	TT	7 (3.8)	3 (4.0)	0.245	1.22 (0.30-4.92)
					TC	45 (24.2)	26 (34.2)		1.65 (0.92-2.96)
					CC	134 (72.0)	47 (61.8)		1
rs8032157	15	q22.31	62267941	*SNX22*	TT	8 (4.3)	5 (6.6)	0.730	1.54 (0.48-4.94)
					TC	65 (34.9)	25 (32.9)		0.94 (0.53-1.68)
					CC	113 (60.8)	46 (60.5)		1
rs442332	16	p13.13	11979625	*SNX29*	GG	1 (0.5)	0 (0.0)	0.791	ND
					GA	20 (10.8)	9 (11.8)		1.11 (0.48-2.56)
					AA	165 (88.7)	67 (88.2)		1
rs8045739	16	p13.13	11985760	*SNX29*	GG	16 (8.6)	3 (4.0)	0.420	0.43 (0.12-1.56)
					GT	63 (34.1)	26 (34.7)		0.95 (0.54-1.69)
					TT	106 (57.3)	46 (61.3)		1
rs7189138	16	p13.13	11990846	*SNX29*	AA	1 (0.5)	0 (0.0)	0.731	ND
					AG	16 (8.7)	8 (10.5)		1.24 (0.51-3.02)
					GG	168 (90.8)	68 (89.5)		1
rs11639821	16	p13.13	12000076	*SNX29*	TT	0 (0.0)	0 (0.0)	0.387	ND
					TC	22 (11.8)	12 (15.8)		1.40 (0.65-2.99)
					CC	164 (88.2)	64 (84.2)		1
rs8045634	16	p13.13	12026752	*SNX29*	GG	6 (3.2)	3 (3.9)	0.945	1.25 (0.30-5.18)
					GT	45 (24.2)	19 (25.0)		1.06 (0.57-1.97)
					TT	135 (72.6)	54 (71.1)		1
rs2865105	16	p13.13	12035270	*SNX29*	AA	2 (1.1)	0 (0.0)	0.656	ND
					AC	18 (9.7)	7 (9.2)		0.94 (0.37-2.34)
					CC	166 (89.2)	69 (90.8)		1
rs8051298	16	p13.13	12045569	*SNX29*	AA	2 (1.1)	0 (0.0)	0.624	ND
					AC	17 (9.1)	6 (7.9)		0.84 (0.32-2.22)
					CC	167 (89.8)	70 (92.1)		1
rs7202124	16	q12.1	49271530	*SNX20*	AA	3 (1.6)	2 (2.6)	0.656	1.57 (0.26-9.63)
					AG	37 (19.9)	12 (15.8)		0.76 (0.37-1.56)
					GG	146 (78.5)	62 (81.6)		1

rs8066529	17	q21.32	43542564	*SNX11*	CC	0 (0.0)	0 (0.0)	0.127	ND
					CT	28 (15.2)	6 (8.1)		0.49 (0.19-1.24)
					TT	156 (84.8)	68 (91.9)		1
rs8080662	17	q21.32	43545818	*SNX11*	CC	4 (2.2)	6 (7.9)	0.042	3.45 (0.93-12.76)
					CT	67 (36.0)	20 (26.3)		0.69 (0.38-1.25)
					TT	115 (61.8)	50 (65.8)		1
rs9890691	17	q21.32	43547935	*SNX11*	GG	4 (2.2)	6 (7.9)	0.042	3.45 (0.93-12.76)
					GT	67 (36.0)	20 (26.3)		0.69 (0.38-1.25)
					TT	115 (61.8)	50 (65.8)		1
rs8071199	17	q21.32	43550334	*SNX11*	AA	4 (2.2)	6 (7.9)	0.042	3.45 (0.93-12.76)
					AT	67 (36.0)	20 (26.3)		0.69 (0.38-1.25)
					TT	115 (61.8)	50 (65.8)		1
rs386155	20	q13.12	43892590	*SNX21*	TT	27 (14.5)	8 (10.5)	0.630	0.65 (0.27-1.58)
					TC	89 (47.9)	36 (47.4)		0.88 (0.50-1.56)
					CC	70 (37.6)	32 (42.1)		1
rs3746495	20	q13.12	43913201	*SNX21*	CC	3 (1.6)	2 (2.6)	0.766	1.60 (0.26-9.79)
					CT	37 (19.9)	13 (17.1)		0.84 (0.42-1.69)
					TT	146 (78.5)	61 (80.3)		1

SNX24 , sorting nexin 24; SNP, single nucleotide polymorphism; CAA, Coronary artery aneurysm; CI, confidence interval; ND, not determined.

p -values were obtained by chi-square test.

Bold, emphasizing statistical significance was considered as *p* value <0.002 (0.05/28).

Table S2 Haplotype distributions of *SNX24* gene SNPs associated with the CAA formation in Taiwanese KD patients

Chromosome	Physical position 1	Physical position 2	SNP1	SNP2	Haplotype	Frequency_CAA+	Frequency_CAA-	Odds ratio	*p* value
5	122228806	122311523	rs154507	rs28891	CC	32.9%	47.6%	0.522	***0.002***
5	122228806	122311523	rs154507	rs28891	GT	45.4%	37.6%	1.41	0.091
5	122228806	122311523	rs154507	rs28891	CT	21.7%	14.8%	1.55	0.066
5	122232671	122311523	rs27740	rs28891	AC	32.9%	47.6%	0.522	***0.002***
5	122232671	122311523	rs27740	rs28891	TT	45.4%	37.6%	1.41	0.091
5	122232671	122311523	rs27740	rs28891	AT	21.7%	14.8%	1.55	0.066
5	122234424	122311523	rs26371	rs28891	TC	32.9%	47.0%	0.551	***0.004***
5	122234424	122311523	rs26371	rs28891	TT	7.9%	3.8%	2.16	0.058
5	122234424	122311523	rs26371	rs28891	CT	59.2%	49.2%	1.58	0.025
5	122234657	122311523	rs6595415	rs28891	CC	32.9%	47.6%	0.522	***0.002***
5	122234657	122311523	rs6595415	rs28891	TT	44.7%	36.6%	1.42	0.079
5	122234657	122311523	rs6595415	rs28891	CT	22.4%	15.9%	1.46	0.098

SNX24, sorting nexin 24; SNP, single nucleotide polymorphism; CAA, Coronary artery aneurysm; CI, confidence interval.

p -values were obtained by chi-square test.

Statistical significance was considered as *p* value <0.0042 (0.05/12).

Table S3 Genotype distributions of *SNX24* gene SNPs in Taiwanese male and female KD patients

SNP	Chromosome	Cytoband	Physical position	Nearest genes		Female	Male		
						No. (%)	No. (%)	*p* value	Odds ratio (95% CI)
rs154507	5	q23.2	122228806	*SNX24*	GG	13 (14.8)	25 (14.4)	0.985	0.99 (0.45-2.21)
					GC	44 (50.0)	89 (51.1)		1.05 (0.59-1.84)
					CC	31 (35.2)	60 (34.5)		1
rs27740	5	q23.2	122232671	*SNX24*	TT	13 (14.8)	25 (14.4)	0.985	0.99 (0.45-2.21)
					TA	44 (50.0)	89 (51.1)		1.05 (0.59-1.84)
					AA	31 (35.2)	60 (34.5)		1
rs26371	5	q23.2	122234424	*SNX24*	TT	18 (20.5)	35 (20.1)	0.629	0.80 (0.37-1.72)
					TC	49 (55.7)	88 (50.6)		0.74 (0.40-1.37)
					CC	21 (23.9)	51 (29.3)		1
rs6595415	5	q23.2	122234657	*SNX24*	TT	12 (13.6)	26 (14.9)	0.948	1.08 (0.48-2.42)
					TC	44 (50.0)	84 (48.3)		0.95 (0.55-1.67)
					CC	32 (36.4)	64 (36.8)		1
rs17149732	5	q23.2	122249811	*SNX24*	TT	12 (13.6)	25 (14.4)	0.980	1.04 (0.46-2.34)
					TG	44 (50.0)	85 (48.9)		0.97 (0.55-1.69)
					GG	32 (36.4)	64 (36.8)		1
rs17149748	5	q23.2	122295266	*SNX24*	CC	12 (13.8)	25 (14.4)	0.940	0.99 (0.44-2.24)
					CT	45 (51.7)	86 (49.4)		0.91 (0.52-1.60)
					TT	30 (34.5)	63 (36.2)		1
rs1038078	5	q23.2	122309550	*SNX24*	AA	12 (13.6)	25 (14.4)	0.941	1.01 (0.45-2.27)
					AG	45 (51.1)	85 (48.9)		0.91 (0.52-1.60)
					GG	31 (35.2)	64 (36.8)		1
rs28891	5	q23.2	122311523	*SNX24*	CC	15 (17.0)	32 (18.4)	0.830	0.99 (0.46-2.14)
					CT	47 (53.4)	86 (49.4)		0.85 (0.47-1.53)
					TT	26 (29.5)	56 (32.2)		1
rs6595423	5	q23.2	122341433	*SNX24*	CC	11 (12.5)	25 (14.4)	0.899	1.14 (0.50-2.60)
					CT	45 (51.1)	85 (48.9)		0.94 (0.54-1.65)
					TT	32 (36.4)	64 (36.8)		1

SNX24 , sorting nexin 24; SNP, single nucleotide polymorphism; CAA, Coronary artery aneurysm; CI, confidence interval.

p -values were obtained by chi-square test.

Statistical significance was considered as *p* value <0.006 (0.05/9).

Table S4 Genotype distributions of *SNX24* gene SNPs in Taiwanese male KD patients

SNP	Chromosome	Cytoband	Physical position	Nearest genes		All male patients	CAA-	CAA+		
						No. (%)	No. (%)	No. (%)	*p* value	Odds ratio (95% CI)
rs154507	5	q23.2	122228806	*SNX24*	GG	25 (14.4)	14 (11.7)	11 (20.4)	0.316	1.99 (0.75-5.24)
					GC	89 (51.1)	63 (52.5)	26 (48.1)		1.04 (0.51-2.15)
					CC	60 (34.5)	43 (35.8)	17 (31.5)		1
rs27740	5	q23.2	122232671	*SNX24*	TT	25 (14.4)	14 (11.7)	11 (20.4)	0.316	1.99 (0.75-5.24)
					TA	89 (51.1)	63 (52.5)	26 (48.1)		1.04 (0.51-2.15)
					AA	60 (34.5)	43 (35.8)	17 (31.5)		1
rs26371	5	q23.2	122234424	*SNX24*	TT	35 (20.1)	25 (20.8)	10 (18.5)	0.521	0.67 (0.27-1.70)
					TC	88 (50.6)	63 (52.5)	25 (46.3)		0.67 (0.32-1.39)
					CC	51 (29.3)	32 (26.7)	19 (35.2)		1
rs6595415	5	q23.2	122234657	*SNX24*	TT	26 (14.9)	14 (11.7)	12 (22.2)	0.187	2.03 (0.79-5.19)
					TC	84 (48.3)	61 (50.8)	23 (42.6)		0.89 (0.43-1.83)
					CC	64 (36.8)	45 (37.5)	19 (35.2)		1
rs17149732	5	q23.2	122249811	*SNX24*	TT	25 (14.4)	14 (11.7)	11 (20.4)	0.312	1.86 (0.72-4.83)
					TG	85 (48.9)	61 (50.8)	24 (44.4)		0.93 (0.46-1.90)
					GG	64 (36.8)	45 (37.5)	19 (35.2)		1
rs17149748	5	q23.2	122295266	*SNX24*	CC	25 (14.4)	14 (11.7)	11 (20.4)	0.317	1.96 (0.75-5.13)
					CT	86 (49.4)	61 (50.8)	25 (46.3)		1.02 (0.50-2.10)
					TT	63 (36.2)	45 (37.5)	18 (33.3)		1
rs1038078	5	q23.2	122309550	*SNX24*	AA	25 (14.4)	14 (11.7)	11 (20.4)	0.312	1.86 (0.72-4.83)
					AG	85 (48.9)	61 (50.8)	24 (44.4)		0.93 (0.46-1.90)
					GG	64 (36.8)	45 (37.5)	19 (35.2)		1
rs28891	5	q23.2	122311523	*SNX24*	CC	32 (18.4)	25 (20.8)	7 (13.0)	0.059	0.37 (0.14-1.01)
					CT	86 (49.4)	63 (52.5)	23 (42.6)		0.49 (0.24-0.99)
					TT	56 (32.2)	32 (26.7)	24 (44.4)		1
rs6595423	5	q23.2	122341433	*SNX24*	CC	25 (14.4)	14 (11.7)	11 (20.4)	0.312	1.86 (0.72-4.83)
					CT	85 (48.9)	61 (50.8)	24 (44.4)		0.93 (0.46-1.90)
					TT	64 (36.8)	45 (37.5)	19 (35.2)		1

SNX24 , sorting nexin 24; SNP, single nucleotide polymorphism; CAA, Coronary artery aneurysm; CI, confidence interval.

p -values were obtained by chi-square test.

Statistical significance was considered as *p* value <0.006 (0.05/9).

Table S5 Genotype distributions of *SNX24* gene SNPs in Taiwanese female KD patients

SNP	Chromosome	Cytoband	Physical position	Nearest genes		All female patients	CAA-	CAA+		
						No. (%)	No. (%)	No. (%)	*p* value	Odds ratio (95% CI)
rs154507	5	q23.2	122228806	*SNX24*	GG	13 (14.8)	10 (15.2)	3 (13.6)	0.112	2.03 (0.38-10.69)
					GC	44 (50.0)	29 (43.9)	15 (68.2)		3.49 (1.03-11.84)
					CC	31 (35.2)	27 (40.9)	4 (18.2)		1
rs27740	5	q23.2	122232671	*SNX24*	TT	13 (14.8)	10 (15.2)	3 (13.6)	0.112	2.03 (0.38-10.69)
					TA	44 (50.0)	29 (43.9)	15 (68.2)		3.49 (1.03-11.84)
					AA	31 (35.2)	27 (40.9)	4 (18.2)		1
rs26371	5	q23.2	122234424	*SNX24*	TT	18 (20.5)	11 (16.7)	7 (31.8)	0.100	6.05 (1.06-34.38)
					TC	49 (55.7)	36 (54.5)	13 (59.1)		3.43 (0.70-16.81)
					CC	21 (23.9)	19 (28.8)	2 (9.1)		1
rs6595415	5	q23.2	122234657	*SNX24*	TT	12 (13.6)	9 (13.6)	3 (13.6)	0.100	2.33 (0.44-12.45)
					TC	44 (50.0)	29 (43.9)	15 (68.2)		3.62 (1.07-12.25)
					CC	32 (36.4)	28 (42.4)	4 (18.2)		1
rs17149732	5	q23.2	122249811	*SNX24*	TT	12 (13.6)	9 (13.6)	3 (13.6)	0.100	2.33 (0.44-12.45)
					TG	44 (50.0)	29 (43.9)	15 (68.2)		3.62 (1.07-12.25)
					GG	32 (36.4)	28 (42.4)	4 (18.2)		1
rs17149748	5	q23.2	122295266	*SNX24*	CC	12 (13.8)	9 (13.8)	3 (13.6)	0.149	2.17 (0.40-11.6)
					CT	45 (51.7)	30 (46.2)	15 (68.2)		3.25 (0.96-11.03)
					TT	30 (34.5)	26 (40.0)	4 (18.2)		1
rs1038078	5	q23.2	122309550	*SNX24*	AA	12 (13.6)	9 (13.6)	3 (13.6)	0.130	2.25 (0.42-12.03)
					AG	45 (51.1)	30 (45.5)	15 (68.2)		3.38 (1.00-11.42)
					GG	31 (35.2)	27 (40.9)	4 (18.2)		1
rs28891	5	q23.2	122311523	*SNX24*	CC	15 (17.0)	14 (21.2)	1 (4.5)	0.072	0.11 (0.01-1.01)
					CT	47 (53.4)	36 (54.5)	11 (50.0)		0.49 (0.17-1.38)
					TT	26 (29.5)	16 (24.2)	10 (45.5)		1
rs6595423	5	q23.2	122341433	*SNX24*	CC	11 (12.5)	9 (13.6)	2 (9.1)	0.060	1.56 (0.24-9.95)
					CT	45 (51.1)	29 (43.9)	16 (72.7)		3.86 (1.15-12.98)
					TT	32 (36.4)	28 (42.4)	4 (18.2)		1

SNX24 , sorting nexin 24; SNP, single nucleotide polymorphism; CAA, Coronary artery aneurysm; CI, confidence interval.

p -values were obtained by chi-square test.

Statistical significance was considered as *p* value <0.006 (0.05/9).
