# Supplementary material for: Sorting nexin 24 genetic variation associates with coronary artery aneurysm severity in Kawasaki disease patients
Source: Cell Biosci. 2013 Nov 22;3:44. doi: 10.1186/2045-3701-3-44 (PMC4176999; doi:10.1186/2045-3701-3-44)
Supplement: Additional file 2: Figure S1 — SNX24 mRNA expression levels in peripheral blood mononuclear cells between the SNX24 SNP (rs28891) genotypes. The relative SNX24 expression was detected by real-time RT-PCR, and expression from individuals with CC + CT genotypes was compared to that from individuals with TT genotypes. The relative expression levels were expressed as SNX24 mRNA/ HPRT mRNA ratio. Figure S2. Single nucleotide polymorphisms (SNPs) of the SNX24 gene used in this study. Above and middle: Genomic location of SNPs present on chromosome 5. Down: Non-coding RNAs mapped to the intron 3 of SNX24 gene. [file 2045-3701-3-44-S2.pptx]

## Slide 1
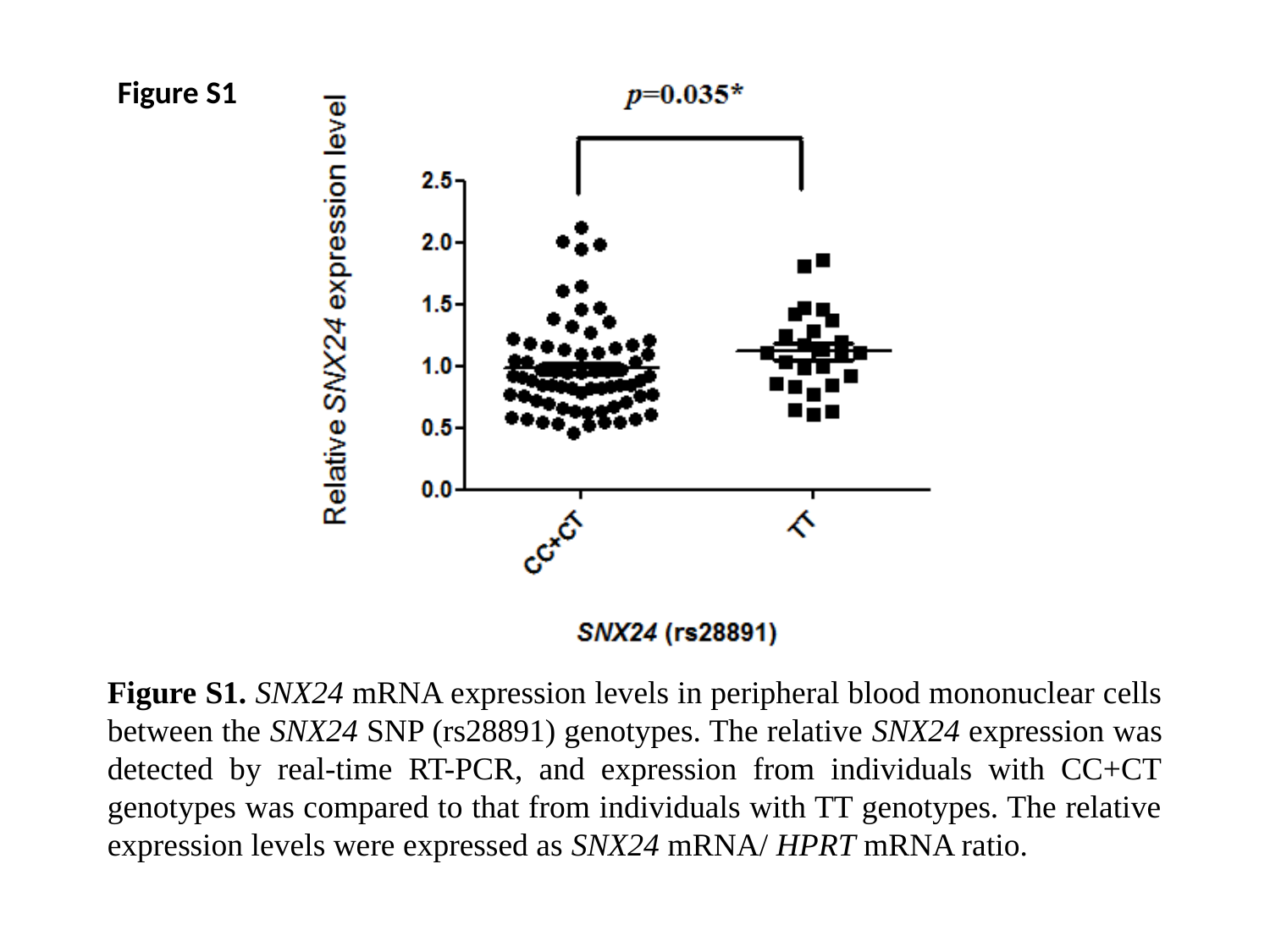

Figure S1
Figure S1. SNX24 mRNA expression levels in peripheral blood mononuclear cells between the SNX24 SNP (rs28891) genotypes. The relative SNX24 expression was detected by real-time RT-PCR, and expression from individuals with CC+CT genotypes was compared to that from individuals with TT genotypes. The relative expression levels were expressed as SNX24 mRNA/ HPRT mRNA ratio.

## Slide 2
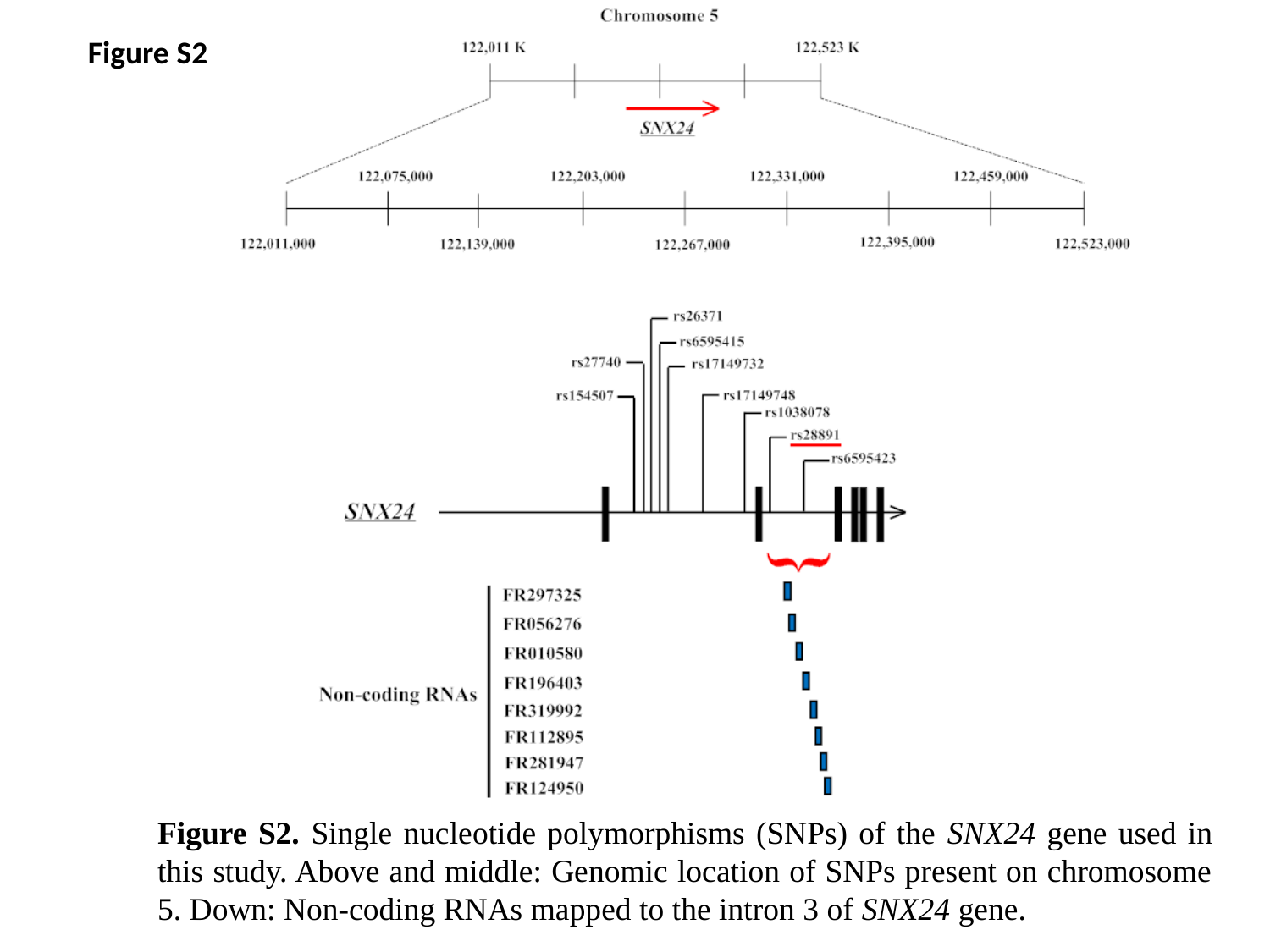

Figure S2
Figure S2. Single nucleotide polymorphisms (SNPs) of the SNX24 gene used in this study. Above and middle: Genomic location of SNPs present on chromosome 5. Down: Non-coding RNAs mapped to the intron 3 of SNX24 gene.
